# Optogenetic stimulation of VTA dopamine neurons reveals that tonic but not phasic patterns of dopamine transmission reduce ethanol self-administration

**DOI:** 10.3389/fnbeh.2013.00173

**Published:** 2013-11-26

**Authors:** Caroline E. Bass, Valentina P. Grinevich, Dominic Gioia, Jonathan D. Day-Brown, Keith D. Bonin, Garret D. Stuber, Jeff L. Weiner, Evgeny A. Budygin

**Affiliations:** ^1^Department of Pharmacology and Toxicology, School of Medicine and Biomedical Sciences, University at BuffaloBuffalo, NY, USA; ^2^Department of Neurobiology and Anatomy, Wake Forest School of Medicine, Winston-SalemNC, USA; ^3^Department of Physiology and Pharmacology, Wake Forest School of Medicine, Winston-SalemNC, USA; ^4^Department of Physics, Wake Forest University, Winston-SalemNC, USA; ^5^Departments of Psychiatry and Cell Biology and Physiology, Neuroscience Center and Bowles Center for Alcohol Studies, The University of North Carolina at Chapel Hill, Chapel HillNC, USA

**Keywords:** dopamine, VTA, nucleus accumbens, optogenetics, ethanol self-administration

## Abstract

There is compelling evidence that acute ethanol exposure stimulates ventral tegmental area (VTA) dopamine cell activity and that VTA-dependent dopamine release in terminal fields within the nucleus accumbens plays an integral role in the regulation of ethanol drinking behaviors. Unfortunately, due to technical limitations, the specific temporal dynamics linking VTA dopamine cell activation and ethanol self-administration are not known. In fact, establishing a causal link between specific patterns of dopamine transmission and ethanol drinking behaviors has proven elusive. Here, we sought to address these gaps in our knowledge using a newly developed viral-mediated gene delivery strategy to selectively express Channelrhodopsin-2 (ChR2) on dopamine cells in the VTA of wild-type rats. We then used this approach to precisely control VTA dopamine transmission during voluntary ethanol drinking sessions. The results confirmed that ChR2 was selectively expressed on VTA dopamine cells and delivery of blue light pulses to the VTA induced dopamine release in accumbal terminal fields with very high temporal and spatial precision. Brief high frequency VTA stimulation induced phasic patterns of dopamine release in the nucleus accumbens. Lower frequency stimulation, applied for longer periods mimicked tonic increases in accumbal dopamine. Notably, using this optogenetic approach in rats engaged in an intermittent ethanol drinking procedure, we found that tonic, but not phasic, stimulation of VTA dopamine cells selectively attenuated ethanol drinking behaviors. Collectively, these data demonstrate the effectiveness of a novel viral targeting strategy that can be used to restrict opsin expression to dopamine cells in standard outbred animals and provide the first causal evidence demonstrating that tonic activation of VTA dopamine neurons selectively decreases ethanol self-administration behaviors.

## Introduction

Alcoholism is a devastating socio-economic problem estimated to account for 4% of the global burden of disease. Despite the widespread use and abuse of alcohol, little is known about the neurocircuitry responsible for the development and progression of alcoholism. Ethanol affects brain function by modulating numerous neurotransmitter and neuromodulator systems, including, but not limited to, GABA (Koob, 2004; Siggins et al., 2005; Weiner and Valenzuela, 2006), glutamate (Woodward, 2000), serotonin (Grant, 1995), norepinephrine (Weinshenker et al., 2000), neuropeptide Y (Thiele et al., 1998), vasopressin (Edwards et al., 2012) adenosine (Nam et al., 2013a,b) and dopamine (Weiss and Porrino, 2002; Gonzales et al., 2004; Koob, 2013). Over the past 20 years, much attention has focused on the mesolimbic dopamine system, which is thought to play an integral role in mediating the positive reinforcing effects of ethanol and other drugs of abuse (Berke and Hyman, 2000; Grace, 2000; Weiss and Porrino, 2002; Gonzales et al., 2004; Stuber et al., 2012). This circuit is comprised of the dopaminergic neurons in the ventral tegmental area (VTA) and their projections to the nucleus accumbens and several other brain regions. A large and growing body of evidence suggests that ethanol acutely enhances mesolimbic dopamine release without significant changes in dopamine transporter function (Budygin et al., 2001; Jones et al., 2006) and that ethanol stimulation of VTA dopamine signaling may contribute to ethanol-drinking behaviors. For example, electrophysiological studies demonstrate that ethanol can directly increase the firing rate of VTA dopamine neurons (Brodie et al., 1999) and elegant microdialysis studies have shown that increases in extracellular dopamine concentrations in the nucleus accumbens are tightly linked to the initial phases of ethanol self-administration (Weiss et al., 1993; Howard et al., 2009; Carrillo and Gonzales, 2011). Pharmacological studies have demonstrated that blockade of dopamine receptors in the nucleus accumbens can significantly decrease operant ethanol self-administration (Czachowski et al., 2001; Samson and Chappell, 2004). However, D2 dopamine receptor antagonists have two distinct actions within the striatum, first they diminish dopamine signaling at the postsynaptic level and secondly these compounds enhance dopamine release through several different presynaptic mechanisms (Wu et al., 2002; Garris et al., 2003; Kita et al., 2007; Park et al., 2010; Belle et al., 2013). Due to these contrasting actions of dopamine antagonists on dopamine dynamics during the time course of ethanol self-administration, it is difficult to relate specific patterns of dopamine transmission to behavioral changes. Furthermore, direct electrical stimulation of the nucleus accumbens in animal models, or deep-brain stimulation (DBS) of the accumbens of alcoholics, reduces ethanol drinking (Kuhn et al., 2007, 2011; Knapp et al., 2009; Muller et al., 2009; Henderson et al., 2010). However, it is possible that DBS, which induces the release of numerous neurotransmitters in the stimulated brain region, accomplishes its effect via non-dopaminergic mechanisms. Therefore, despite many compelling findings, several questions regarding the role of dopamine signaling in the regulation of ethanol drinking remain unanswered. For example, can DBS-induced changes in mesolimbic dopamine transmission alone be responsible for these alterations in alcohol drinking behaviors? If so, which patterns of VTA-accumbal dopamine transmission are responsible for the inhibition and triggering of ethanol seeking and drinking behaviors?

Until recently it has been impossible to parse the causal role of specific neurotransmitter systems in the regulation of ethanol drinking behaviors. Optogenetics provides a means to experimentally control the activation of specific neuronal sub-populations in heterogeneous brain regions where multiple neuronal subtypes exist and to do so with exquisite temporal precision. While several transgenic strategies have been developed to target opsins to dopaminergic neurons in rats and mice, we have developed a novel viral vector approach that can theoretically be used in any rodent species or strain. In this approach, the expression of channelrhodopsin-2 (ChR2) is driven by a tyrosine hydroxylase (TH) promoter, which has previously been shown to restrict expression primarily to dopaminergic neurons (Oh et al., 2009).

Here, we injected an adeno-associated virus (AAV) construct, in which ChR2 expression is restricted to TH-positive neurons into the rat VTA and characterized the effect of tonic and phasic activation of VTA dopaminergic neurons on dopamine release in the nucleus accumbens and ethanol drinking behaviors. Our data confirm that the viral strategy selectively targets ChR2 expression to dopamine neurons within the VTA and that low (5 Hz) and high (50 Hz) frequency stimulation of VTA dopamine neurons results in distinct patterns of accumbal dopamine release. Moreover, ethanol drinking behaviors were selectively reduced by tonic, but not phasic, VTA dopamine release. Collectively, these data demonstrate a novel strategy that successfully targets opsins to VTA dopamine cells in standard outbred rats and provide new insight in the specific temporal dynamics of dopamine signaling that regulate ethanol drinking behaviors.

## Materials and methods

### Viral packaging

Viruses were packaged using a standard triple transfection packaging protocol to generate pseudotyped AAV2/10 (Xiao et al., 1998). The three plasmids consisted of an AAV2 plasmid containing the transgene to be packaged, pHelper (Stratagene, La Jolla, CA) which contributed the necessary adenoviral helper functions, and an AAV2/10 rep/cap plasmid that provides the AAV2 replicase and AAV10 capsid genes (Gao et al., 2002; De et al., 2006). Dr. K. Deisseroth (Stanford University) kindly provided the ChR2-EYFP construct while the TH promoter (Oh et al., 2009) was obtained from Dr. K-S. Kim (McLean Hospital). Virus packaging and titering has been described previously (Bass et al., 2010).

### Stereotaxic virus injection

Naive male Long-Evans rats were anesthetized with ketamine hydrochloride (100 mg/kg, i.p.) and xylazine hydrochloride (20 mg/kg, i.p.) and placed in a stereotaxic frame. The scalp was shaved, swabbed with iodine and a central incision made to expose the skull. Two small holes were drilled and 2 skull screws were placed in to secure a cement cap. A third hole was drilled above the right VTA (from bregma: anterior-posterior, 5.8 mm; lateral, 0.7 mm) and an optic-fluid cannula (OFC) (Doric Lenses, Canada) was implanted (dorsal-ventral, 7.3 mm). Finally, 1.3 μl of virus that expresses ChR2-EYFP with expression driven by a TH promoter was slowly injected into the VTA (dorsal-ventral, 7.3 mm) over 3 min via a Hamilton syringe connected to the OFC. The exposed skull was coated with dental cement secured by skull screws and upon drying the animals were returned to their home cages for recovery.

### Ethanol self-administration

Ethanol drinking was assessed using an intermittent home-cage drinking procedure which we and others have previously shown to engender relatively high levels of ethanol intake in male Long-Evans rats (Wise, 1973; Simms et al., 2008). Briefly, subjects were given access to 20% ethanol (v/v) and water for 24 h periods every Monday, Wednesday, and Friday with only water available on the remaining days. Water and ethanol were given in graduated drinking tubes (MED Associates, St. Albans, VT, USA), and the position of the bottles was alternated on each drinking day to control for potential side preferences. During ethanol sessions, water and ethanol consumption were measured daily after 30 min and 24 h (daily) access to ethanol. Prior data from our lab have shown that brain ethanol concentrations at the 30 min time point in this model (in rats showing similar intake levels) approximate 40 mg/dl (Chappell et al., 2013). The latency to the first lick and total number of licks during the first 30 min of each drinking session were also measured using custom-made lickometers.

### Voltammetric recordings

For the evaluation of changes in extracellular dopamine concentrations in response to optical stimulation of the VTA, rats were anesthetized with urethane (1.5 g/kg, i.p.) and placed in a stereotaxic frame. The cement above the striatum was removed and a hole for a carbon fiber electrode (~100 μm in length, 6 μm in diameter) insertion was drilled (from bregma: anterior-posterior, 1.2 mm; lateral, 2 mm). An Ag/AgCl reference electrode was implanted in the contralateral hemisphere and a carbon fiber electrode was positioned in the striatum (dorsal-ventral, 4.2–7.2 mm). An optic-fluid cannula (Doric Lenses, Canada) with fiber 200 μm in diameter (dorsal-ventral, 7.3 mm) was connected to the laser (Viasho, China). The reference and carbon fiber electrodes were connected to a voltammetric amplifier (UNC Electronics Design Facility, Chapel Hill, NC) and voltammetric recordings were made at the carbon fiber electrode every 100 ms by applying a triangular waveform (−0.4 to +1.3 V, 300 V/s). Data were digitized (National Instruments, Austin, TX) and stored on a computer. Light evoked dopamine release was identified by the background-subtracted cyclic voltammogram. Carbon fiber microelectrodes were calibrated *in vitro* with known concentrations of dopamine (2–5 μM). Dopamine uptake was determined from the clearance rate of dopamine and was assumed to follow Michaelis-Menten kinetics. The changes in dopamine during and after optical or electrical stimulation were fit using the equation:

$$d[DA]/dt\;=(f){\left[DA\right]}_{p}-\left(V_{\text{max}}/\{\left(K_{m}/[DA]\right)+1\}\right)$$

where *f* is the stimulation frequency (Hz), [*DA*]_p_ is the concentration of dopamine released per stimulus pulse, and *V*_max_ is the maximal rate of dopamine uptake, which is proportional to the number of available DAT proteins. The baseline value of *K*_m_ was calculated to be between 0.16–0.2 μM, a value determined in rat brain synaptosomes and commonly used in the analysis of voltammetric data (Near et al., 1988; Garris and Rebec, 2002). The derivative form of the above equation was used to simulate the dopamine response (Garris and Rebec, 2002; Phillips et al., 2003a; Oleson et al., 2009; Pattison et al., 2011, 2012).

### Optical delivery

The optical setup consisted of a laser at wavelength 473 nm (Beijing Viasho Technology Co., Ltd, Beijing, China) with a maximum power output of 100 mW. The laser was modulated using the TTL input control port on the laser power supply with the control signals provided by a programmable function generator (Hewlett-Packard model 8116A). The start of the whole optical pulse procedure was initiated by manually firing a pulse generator (Systron Donner Model 100C) that then triggered a digital delay generator (SRS Model DG535) that was used to gate the function generator. The main purpose of the digital delay generator was to make sure that the function generator was properly gated to only select out a finite number of pulses from the continuous waveforms normally produced by the function generator. The function generator would produce a series of either 5 Hz or 50 Hz square pulses, and the total number of pulses in one data stream (50 and 250 respectively) would be controlled by the digital delay generator, since the temporal length of a gate pulse sent to the function generator would determine the number of square pulses the function generator would produce for each trigger. Each pulse within a series of pulses had a temporal width of 4 ms. The laser power output was measured using a commercial power meter (Newport Model 1815C). Synchronization of the laser to the voltammetric recording was achieved by manually triggering the laser 5 s after initiating the voltammetric recording that was controlled through an electronic controller instrument that was built by the Electronics Design Facility at the University of North Carolina at Chapel Hill. The light paradigm was 50 light pulses at 50 Hz with 30 s intervals for phasic stimulation and 250 light pulses at 5 Hz with 1 s intervals for tonic stimulation.

### Immunohistochemistry

Rats were deeply anesthetized with a mixture of ketamine (100 mg/kg) and xylazine (100 mg/kg) and transcardially perfused with 10% normal buffered formalin. Brains were removed and incubated overnight with fix at 4°C and then transferred and incubated again overnight in 25% sucrose until the brains sank. They were sectioned at 50 microns on a dry ice chilled stage using an American Optical 860 sliding microtome. Floating coronal sections containing the midbrain were processed for immunohistochemistry. Briefly, sections were rinsed in PBS for 5 min, and again in PBS + 0.5% triton X-100 three times for 10 min. Sections were incubated with primary antibody overnight at 4°C while shaking. Primary antibodies consisted of a mouse anti-tyrosine hydroxylase (ImmunoStar #22941) at a 1:4000 dilution and a rabbit anti-GFP (*Invitrogen* #A6455) at 1:2000. All antibodies were diluted in PBS + 0.3% triton X-100. The next day the sections were rinsed 3 times for 10 min each in PBS and then incubated with secondary antibodies consisting of Alexa 555 donkey anti-mouse (*Invitrogen* #A31570, 1:4000) and Alexa 488 goat anti-rabbit (*Invitrogen* #A11034, 1:2000) for 2 h at room temperature while shaking. Sections were rinsed again in PBS three times for 10 min, then mounted on slides and coverslipped using Prolong Gold media. Slides were visualized on a Zeiss Axio Observer Confocal microscope.

### Statistical analysis

Data were analyzed in GraphPad Prism (GraphPad Software, San Diego, CA). A two-way ANOVA was performed for the neurochemical studies characterizing differences between the effect of tonic and phasic light activation on accumbal dopamine efflux and for the effect of these stimulation patterns on the number of ethanol and water licks. The effects of optical stimulations on other ethanol drinking parameters (e.g., intake, latency to first lick) were analyzed by one-way analysis of variance. Bonferroni's tests were used for all *post-hoc* comparisons. Pearson correlation analysis was used to evaluate the relationship between the number of ethanol licks and the amount of ethanol consumed (g/kg). The data are presented as mean ± SEM and the criterion for significance was set at *P* < 0.05.

## Results

### Targeting ChR2 to VTA dopaminergic neurons in wild-type rats

**Figure 1:**
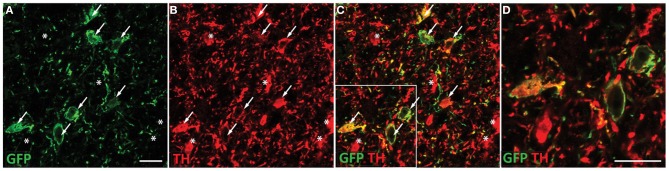
**ChR2-EYFP expression is targeted to dopaminergic neurons in the VTA. Coronal section containing the midbrain of a representative rat injected with TH restricted ChR2 AAVs were co-stained with EYFP and TH antibodies**. TH staining was apparent throughout the VTA and substantia nigra. Robust EYFP expression was observed in the VTA only. EYFP immunohistochemical staining revealed multiple ChR2 positive cell bodies present **(A)**. Tyrosine hydroxylase co-staining **(B)** demonstrated many TH positive neurons in the VTA, which colocalized with the ChR2 signal **(C)**. A portion of the merged image is magnified in **(D)**. Neurons that express both TH and ChR2 are marked with arrows while neurons that are TH positive only (no apparent ChR2) are marked with asterisks. ChR2 was not observed in non-TH positive neurons. Scale bar equals 20 microns.

To determine the contribution of VTA dopaminergic neurotransmission to alcohol seeking behavior, we generated AAVs that target dopaminergic neurons in the VTA. A TH promoter was used to restrict expression of ChR2 to TH-positive neurons. When injected directly into the VTA, expression was restricted to TH-positive neurons as revealed by immunohistochemical staining (Figure 1). Robust TH expression was observed throughout the VTA and substantia nigra, however, ChR2 expression was found only on cell bodies in VTA in sections containing the entire midbrain. Co-staining revealed many TH-positive cells (Figure 1B) that also expressed ChR2 (Figures 1C,D). We did not observe ChR2 expression on VTA cells that did not express TH (Figures 1C,D). However, there were some apparent TH-positive cells that did not express observable levels of ChR2. This pattern of expression is expected from viral delivery in that not all neurons in a given virus infusion site will be transduced, or receive equivalent DNA payloads.

### Voltammetric assessment of optically-evoked dopamine release in rat nucleus accumbens

**Figure 2:**
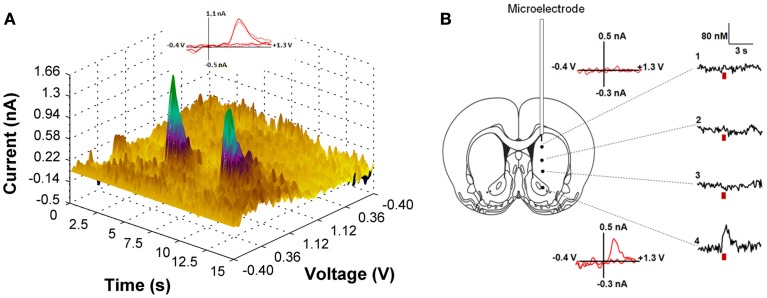
**(A)** Light-induced dopamine release in the ventral striatum. The left panel demonstrates a three dimensional color plot topographically depicting voltammetric data collected with FSCV in the nucleus accumbens of a single anesthetized rat before, during and after optical stimulation of the VTA. Green spikes represent dopamine transients triggered by 30 Hz, 30 pulses light stimulation with 5 s intervals between pulse-train groups. Inset: Background-subtracted cyclic voltammograms taken at the end of the light stimulation indicate that the change in current is due to dopamine oxidation (solid line—for the first dopamine spike; dotted line—for the second spike). **(B)** Optically-evoked dopamine concentrations in different regions of the striatum from one representative animal are presented. The fiber optic was fixed in the VTA (anterior-posterior, 5.8 mm; lateral, 0.7 mm; dorsal-ventral, 7.3 mm), while the voltammetric carbon fiber electrode was lowered to various depths throughout the dorsal and ventral striatum to determine level of dopamine release (anterior-posterior, 1.2 mm; lateral, 2.0 mm and dorsal-ventral varied with 1: 4.2 mm; 2: 5.2 mm; 3: 6.2 mm; 4: 7.2 mm). Red bar indicates the time of light stimulation. Background-subtracted cyclic voltammograms from 1 and 4 are also presented.

We first employed *in vivo* fast-scan cyclic voltammetry (FSCV) to confirm that the level of ChR2 expression achieved in the VTA is sufficient for optical stimulation of dopamine release in the rat nucleus accumbens. Figure 2A demonstrates accumbal dopamine transients, triggered by blue-light stimulation of the VTA (30 Hz, 30 flashes with 4-ms flash length, 5.7 mW) in a single animal. The rising fractions of dopamine effluxes were time-locked to the 1-s blue pulse, whereas dopamine concentrations rapidly declined immediately at the end of the pulse. Background-subtracted voltammograms taken at the peak of stimulation confirmed that the signal detected is dopamine. No other electrochemical activity changes were observed during the period of detection. Notably, the viral targeting strategy we employed resulted in expression of ChR2 on dopaminergic terminals in VTA projection areas, such as the nucleus accumbens. As demonstrated in Figure 2B, robust light-activated dopamine signals could be consistently recorded in the nucleus accumbens, however when the recording electrode was moved to regions within the dorsal striatum, which receives rich dopaminergic innervation from the substantia nigra, no measurable dopamine responses were evoked. To calculate the parameters of dopamine release and uptake, light-evoked changes in dopamine concentration were modeled as a balance between release and uptake (Wightman et al., 1988; Wu et al., 2002; Phillips et al., 2003a). The magnitudes of dopamine uptake parameters, including maximal velocity of dopamine reuptake rate or *V*_max_ and apparent *K*_m_ (Michaelis-Menten constant) were indistinguishable from those previously reported for the rat nucleus accumbens using FSCV *in vitro* and *in vivo* (Phillips et al., 2003a; Jones et al., 2006; Oleson et al., 2009; Pattison et al., 2011, 2012). *V*_max_ and *K*_m_ were 2420 ± 314 nM/s and 176 ± 10 nM, respectively. The average magnitude of dopamine efflux per blue-light pulse or per flash (*DA*_p_f) was 24 ± 6 nM, which is less than the amount dopamine released by an electrical pulse of the same length (4 ms).

**Figure 3:**
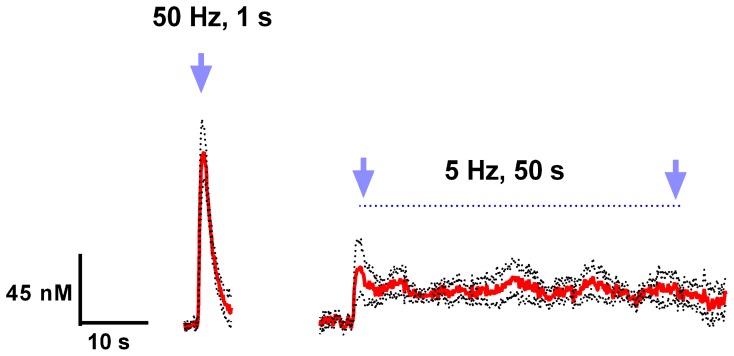
**Light activation of VTA dopaminergic neurons can mimic phasic and tonic dopamine release**. Average dopamine concentration changes recorded in rat nucleus accumbens were evoked by 50 Hz, 50 pulses, and 5 Hz, 250 pulses (4 ms pulse width) optical stimulation of the VTA. Dopamine was identified by its oxidation (≈0.6 V) and reduction (≈−0.2 V on the negative going scan) features. These data are presented as a mean ± s.e.m. denoted by red solid and black broken lines, respectively (*n* = 5).

Finally, we examined various parameters of optical stimulation of the VTA, which allows us to induce phasic (a relatively large but transient rise of extracellular dopamine concentration) and tonic (relatively low but sustained dopamine increase) release patterns in the nucleus accumbens. We found that high frequency (50 Hz), brief (1s) stimulation could mimic phasic dopamine responses, while low frequency (5 Hz), long lasting (50 s) stimulation resulted in a tonic elevation in dopamine concentration (Figure 3). As expected, there was a significant difference between these two contrasting dopamine responses [*F*_(1, 782)_ = 71.21; *p* < 0.0001]. Changes in dopamine concentration over time were also significantly different [*F*_(98, 782)_ = 14.38; *p* < 0.0001].

### Effect of tonic and phasic dopamine release on ethanol drinking behaviors

The overall goal of this study was to determine the relationship between accumbal dopamine release patterns and ethanol drinking behaviors. To that end, we first exposed eight rats to an intermittent drinking procedure for 7 weeks. This protocol engenders relatively high levels of ethanol intake and importantly, subjects consume approximately 25% of their daily ethanol intake in the first 30 min of each drinking session, consistently achieving blood ethanol levels between 40–80 mg % (Simms et al., 2008). Rats that demonstrated high and relatively stable levels of ethanol intake during 7 weeks of baseline drinking were then injected with the viral construct (*n* = 5). High drinking rats were selected for these initial studies to explore the effects of optogenetic manipulation of the VTA on the voluntary consumption of pharmacologically meaningful doses of ethanol (Samson and Czachowski, 2003).

**Figure 4:**
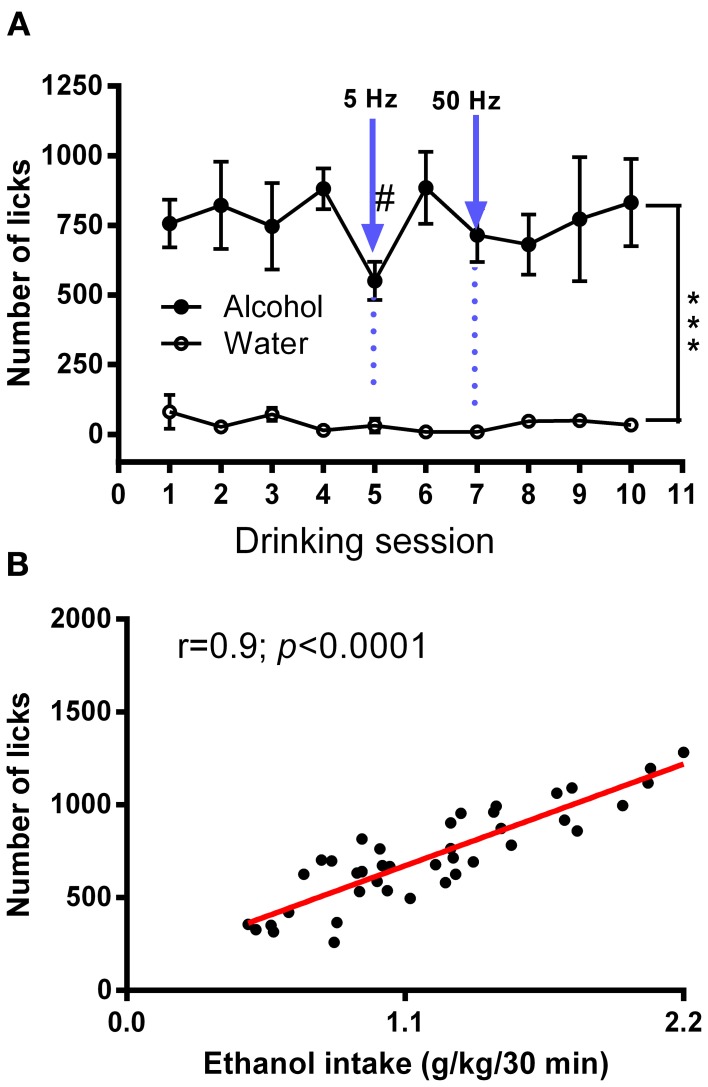
**Average numbers of licks for ethanol and water in an intermittent 2-bottle choice drinking assay with optical stimulation. (A)** Average daily ethanol (20%) and water licks during 10 drinking sessions was measured. There was a significant difference in the number of licks between ethanol and water [*F*_(1, 72)_ = 55.29; ^***^P < 0.0001). The effects of tonic (5 Hz) and phasic (50 Hz) optical stimulation of VTA dopaminergic neurons applied during the first 10 min of the 5 and 7th session, respectively, were explored. No significant changes in the number of water licks was found, while there was a considerable trend (*t* = 2.4) toward a decrease in ethanol licking following 5-Hz stimulation. Data are presented as a mean ± SEM (*n* = 5). **(B)** Relationship between ethanol dose (g/kg) and total number of licks obtained during a 30 min drinking session. There was a strong, positive correlation between the number of ethanol licks and the amount of ethanol consumed (Pearson *r* = 0.9; *p* < 0.0001). The measures were taking from multiple sessions.

**Figure 5:**
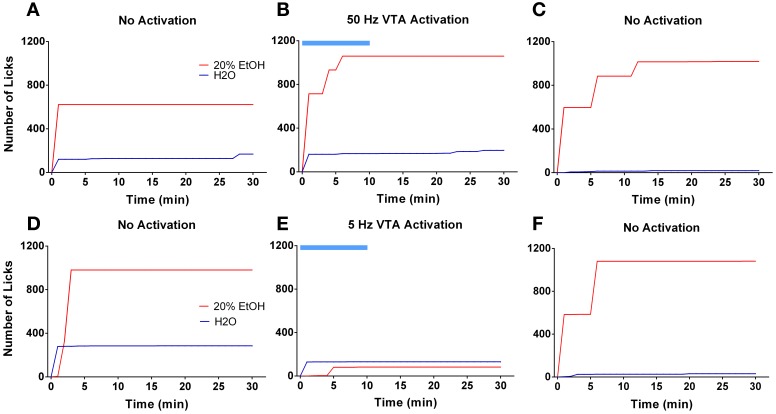
**Tonic but not phasic dopamine release reduces ethanol self-administration**. Graphs demonstrate representative cumulative records of licking during consecutive 30 min drinking sessions, where 20% ethanol solution and water are available. The top panel demonstrates ethanol and water drinking patterns of a single rat during 3 separate sessions that were performed 2 days apart: **(A,C)** two sessions were with no stimulation; **(B)** one session was with optical stimulation of the VTA at 50 Hz frequency. The bottom panel shows drinking patterns from analogous sessions with 5 Hz frequency stimulation: **(D,F)** two sessions were with no stimulation; **(E)** one session with VTA stimulation at 5 Hz frequency. Optogenetic activation of VTA dopamine neurons at a low (5 Hz) but not high (50 Hz) frequency affects ethanol drinking behavior. The blue bar indicates a time of the stimulation.

**Figure 6:**
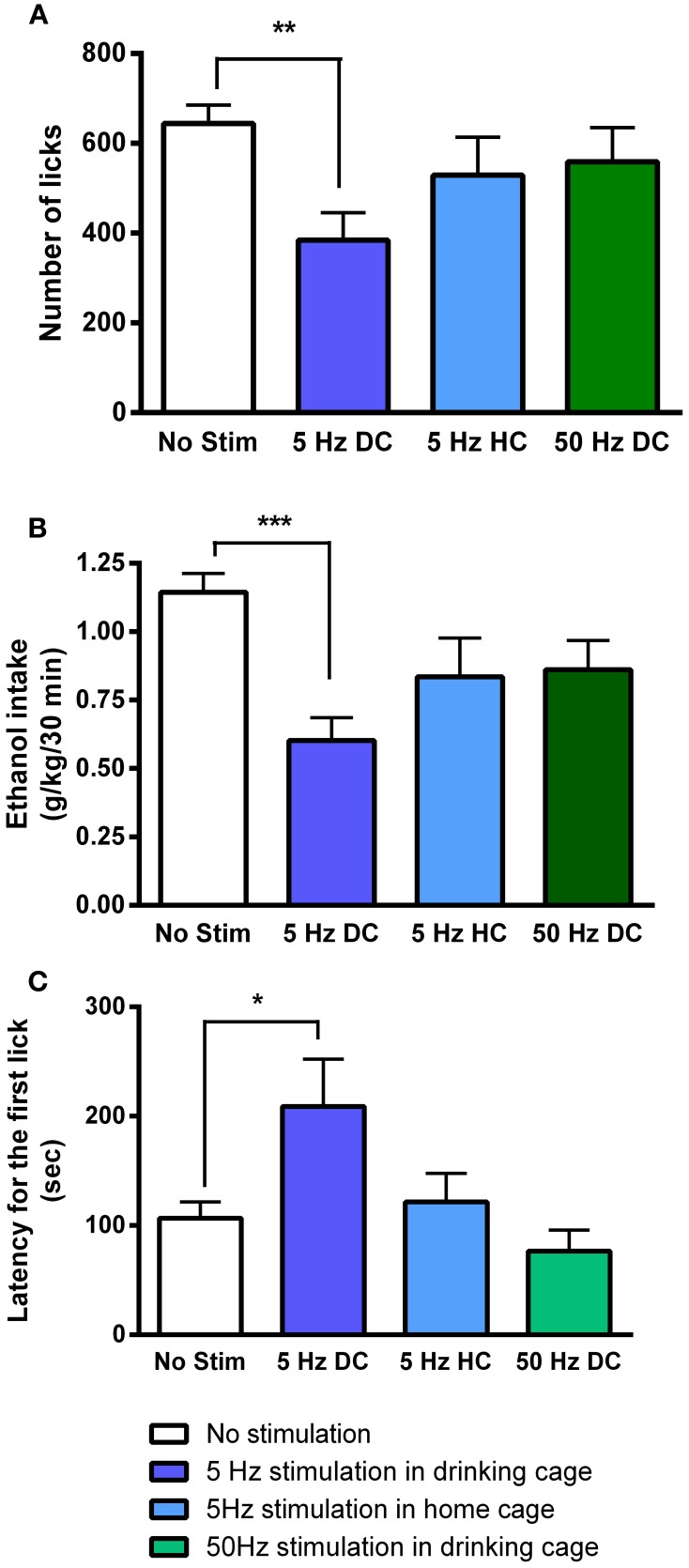
**Tonic dopamine release alters ethanol drinking measures only when optogenetic stimulation is applied in the drinking cage**. Bar graphs illustrate averaged values of **(A)** number of licks, **(B)** total dose of ethanol consumed (g/kg), and **(C)** latency for the first lick (s) across multiple sessions. The sessions were performed in the drinking cage (DC) with no stimulation (No Stim), with 5-Hz (5 Hz DC) and with 50-Hz stimulation (50 Hz DC), applied in the first 10 min, and in the home cage (HC, 10 min immediately prior to being placed in the drinking cage) with 5-Hz stimulation (5 Hz HC). The effect of 5-Hz stimulation applied in the drinking cage compared to No Stim was significant for all drinking parameters. ^*^P < 0.05, ^**^P < 0.01, ^***^P < 0.001 compared with the session when no stimulation was applied.

Ten days following the injection, ethanol intake returned to baseline levels. Figure 4A shows licking patterns of ethanol and water during the first 30 min of 10 drinking sessions. As observed in previous studies, subjects robustly preferred ethanol to water as indicated by a significant difference in the number of licks between ethanol and water [*F*_(1, 72)_ = 55.29; *p* < 0.0001]. Taking advantage of the stable drinking patterns, we explored the effects of tonic and phasic optical stimulation of dopaminergic neurons in the VTA on drinking behaviors (5 Hz and 50 Hz stimulations during the first 10 min of the 5 and 7th session, respectively). No significant change (*p* > 0.05) in the number of water licks was found, while a noticeable trend (*t* = 2.4) toward a decrease in ethanol licking following 5 Hz stimulation was evident (Figure 4A). As expected, there was a strong, positive correlation between the number of ethanol licks and the amount of ethanol consumed (Pearson *r* = 0.9; *p* < 0.0001, Figure 4B). We further explored the effects of optogenetic stimulation of VTA TH-positive neurons on drinking, using a larger group of animals (*n* = 8) in which optical stimulation was performed in a randomized order. This design allowed us to avoid possible confounds such as a habituation. In this larger cohort of animals, tonic activation of VTA dopamine cell bodies significantly affects ethanol drinking behaviors. Figure 5 demonstrates representative cumulative records of licking for a single rat on different drinking days. We observed a decrease in ethanol consumption measured as the number of licks (Figure 6A, 60 ± 16 % decrease from control, no stimulation, *t* = 3.45; *p* < 0.01) and the amount of ethanol consumed (Figure 6B, 53 ± 13 %, *t* = 4.42; *p* < 0.001), when rats were stimulated at 5 Hz in the drinking cage. In marked contrast, phasic stimulation of VTA dopamine cells during the first 10 min of the drinking session had no effect on any measures of ethanol intake. Moreover, no significant changes in ethanol intake measures were observed when subjects received tonic stimulation in their home cages just prior to the drinking sessions (*p* > 0.05). Finally, we found a significant two-fold delay for the first ethanol lick when rats were stimulated with the frequency that induces tonic dopamine release (Figure 6C, *t* = 3.08; *p* < 0.05). No significant changes in this parameter were evident during other stimulating protocols.

## Discussion

It is increasingly clear that revealing the precise contribution of dopamine neurons to specific behaviors requires more sophisticated control over their activity than traditional techniques can provide (Tsai et al., 2009; Steinberg and Janak, 2012; Tye et al., 2012; van Zessen et al., 2012; Steinberg et al., 2013). Here, we employed newly developed optogenetic tools to address critical questions regarding the role of dopamine in alcohol drinking-related behaviors. A viral-mediated gene delivery strategy was used to selectively express ChR2 on dopamine cells in the VTA of wild-type rats. We then used this approach to manipulate mesolimbic dopamine transmission during ethanol drinking sessions. The results confirmed that optogenetic activation of VTA dopamine neurons can induce dopamine release in accumbal terminal fields with very high temporal and spatial precision. Brief (1 s) high-frequency (50 Hz) VTA stimulation induced phasic patterns of dopamine release, which were characterized by a relatively large (~100 nM) and transient rise of extracellular dopamine concentration in the nucleus accumbens. Lower frequency stimulation (5 Hz) for longer periods (50 s), mimicked tonic dopamine increases, characterized by relatively low (~40 nM), but longer-lasting elevations in accumbal dopamine levels. Finally, combining this approach with an intermittent ethanol drinking procedure, we found that forcing mesolimbic dopamine transmission into a tonic but not phasic mode significantly affects ethanol drinking measures while having no effect on water intake.

Although several elegant methods have been developed that achieve selective expression of opsins in dopamine cells, all of these methods are restricted to specific lines of genetically engineered rats and mice. A major goal of this study was to develop an alternative targeting strategy that could theoretically work in any animal. To that end, we used a previously characterized rat TH promoter to restrict the expression of ChR2. By infusing the virus directly into the VTA, we restrict the expression to dopaminergic neurons in this region only. Our *in vivo* neurochemical data demonstrate the effectiveness of this approach in a standard outbred wild-type rat strain. Optical stimulation of the VTA induced reliable dopamine efflux in the nucleus accumbens core with release dynamics consistent with previously published uptake and release values for this brain region. In addition, exquisite temporal and spatial control of dopamine release was achieved. High frequency, phasic stimulation resulted in large, rapid dopamine transients whereas longer duration, tonic stimulation evoked smaller but more sustained patterns of dopamine release. Importantly, dopamine release was restricted to VTA terminal fields in the nucleus accumbens. No detectable dopamine transients were observed when the recording electrode was moved to the dorsal striatum, which does not receive appreciable dopamine innervations from the VTA. In a previous study, where a non-selective AAV was used to express ChR2 in the substantia nigra, which preferentially innervates the dorsal, but not ventral striatum, similar spatial precision was achieved (Bass et al., 2010). Optical activation of the SN evoked dopamine transients in the dorsal striatum but not the nucleus accumbens. Dopamine neurons in the VTA and SN have distinct roles in various behavioral phenomena including motivation, reinforcement and learning (Steinberg and Janak, 2012; Stuber et al., 2012; Watabe-Uchida et al., 2012). Therefore, the ability to control dopamine release specifically, without induction of other neurotransmitters, in anatomically distinct terminal fields is crucial. The activation of both of these dopamine circuits using traditional electrical or pharmacological approaches may result in different (and more complex) behavioral consequences, and precludes our ability to establish a causal relationship between dopamine release in distinct brain regions and behavior changes. Importantly, although the VTA sends a robust dopamine projection to the nucleus accumbens, it also projects to the amygdala, prefrontal cortex and hippocampus (Beckstead et al., 1979; Swanson, 1982), which are also likely involved in the control of ethanol drinking. Optical stimulation of the VTA undoubtedly triggers dopamine efflux in these other brain areas, although the present study did not assess dopamine efflux in these regions. Thus, although these data provide causal evidence that VTA dopamine neurons directly modulate voluntary ethanol drinking behaviors, further studies will be needed to establish whether VTA dopamine terminal fields in the nucleus accumbens alone play a causal role in ethanol drinking behaviors.

It is well known that dopamine neurons exhibit two main firing modes: single spikes at a frequency of ~5 Hz and bursts of several action potentials at higher frequencies (≥20 Hz) (Grace and Bunney, 1983; Schultz et al., 1993; Anstrom et al., 2009). These firing patterns generate distinct tonic and phasic patterns of dopamine release, resulting in dopamine concentration fluctuations on a time scale of several minutes and seconds, respectively (Wightman and Robinson, 2002; Owesson-White et al., 2012). These patterns appear to subserve different functions that result in distinct behavioral consequences. Our results clearly demonstrate that both phasic and tonic patterns of dopamine release can be reproduced in the rat nucleus accumbens by optical stimulation of the VTA. These dopamine increases were time-locked to the light stimulation and their amplitudes were within the range seen with natural dopamine fluctuations as measured by voltammetry.

Dopamine neurotransmission in the nucleus accumbens contributes considerably to many vital behaviors, yet the nature of this contribution is a matter of debate (Salamone et al., 1997; Phillips et al., 2003b; Robbins and Everitt, 2007; Bromberg-Martin et al., 2010; Nicola, 2010; Steinberg et al., 2013). One prominent idea is that increases in dopamine release in this brain region are essential for the activation of reward-seeking for both natural rewards as well as abused substances, like alcohol. Although extensive and compelling studies have demonstrated increases in accumbal extracellular dopamine during ethanol self-administration and during the anticipatory period preceding ethanol availability (Weiss et al., 1993; Howard et al., 2009; Carrillo and Gonzales, 2011), it has been a challenge to establish whether specific patterns of dopamine transmission play a causal role in the initiation or suppression of ethanol seeking and consumption. Here, we provide clear evidence that ethanol drinking can be selectively influenced by distinct patterns of VTA dopamine cell activation. We found a significant delay to the first ethanol lick and a decrease in the total amount of ethanol consumed when VTA dopamine cell bodies were activated at a low frequency (5 Hz). Notably, high-frequency stimulation had no effect on these drinking measures. Low-frequency stimulation of VTA dopamine cells shifts accumbal dopamine release activity to tonic mode, characterized by a small but prolonged increase in extracellular dopamine levels. These changes have been suggested to reduce phasic dopamine release through a D2 dopamine autoreceptor–mediated feedback mechanism (Grace, 2000; Phillips et al., 2003b; Oleson et al., 2009). In fact, it has been proposed that phasic dopamine is required for the ability of reward predictive stimuli to motivate behavioral responding directed toward obtaining the reward (Steinberg et al., 2013).

Notably, the intermittent-access drinking procedure employed in this study promotes binge-like ethanol drinking during the first 5–10 min of each session where ethanol is available, coincident with the placement of animals in the drinking chamber along with the presentation of drinking-associated cues. We hypothesize that optogenetically forcing dopamine release into a tonic mode may prevent phasic dopamine signals normally triggered by the contextual cues associated with the drinking chamber, therefore decreasing ethanol intake during the stimulation period. Notably, ethanol remains available for the last 20 min of each session, providing subjects with the opportunity to compensate for the amount of ethanol that was not consumed during the first 10 min. However, when optical stimulation ceased, rats did not attempt to compensate for their loss. This may be due the fact that the tonic increase in accumbal dopamine concentration may mimic the pharmacological effect of alcohol. In this case, rats would require less ethanol to perceive the normal intoxicating effects of this drug. However, tonic optical stimulation delivered in the home cage, during the 10 min preceding ethanol drinking sessions, had no effect on ethanol consumption or the pattern of intake. Therefore, it seems more likely that optogenetic suppression of cue-induced phasic dopamine release associated with the onset of a drinking session may diminish drinking behaviors even after the optogenetic stimulation ceases.

High-frequency (50 Hz) optogenetic activation of VTA dopamine cells can induce measurable phasic dopamine response in the mouse nucleus accumbens and is sufficient to induce conditional place preference (Tsai et al., 2009). Moreover, high-frequency (60 Hz) electrical stimulation of the rat VTA also induces phasic dopamine release and this pattern of dopamine cell activation promotes the initiation of seeking behavior in rats self-administering cocaine (Phillips et al., 2003b). In addition, phasic activation of VTA dopamine neurons in mice has been reported to completely reverse chronic mild stress-induced anhedonia, significantly increasing sucrose intake (Tye et al., 2012). Therefore, we expected that phasic stimulation would increase ethanol drinking measures. Surprisingly, in our study no significant changes in the latency to the first ethanol drink or the total amount of ethanol consumed were seen. One possible reason for this finding is that we employed rats that were already habituated to the intermittent drinking procedure and also exhibited relatively high levels of ethanol intake at the time when optogenetic stimulation was delivered. Therefore, the inability of phasic stimulation to enhance ethanol drinking measures may have resulted from a ceiling effect. In other words, since these rats were likely already highly motivated to consume ethanol, perhaps additional optogenetic enhancement of phasic dopamine release could not further increase ethanol intake.

Previous studies have found that appetitive (or motivational) and consummatory measures of ethanol drinking are not correlated (Samson and Czachowski, 2003) and are likely mediated by distinct, albeit related, neural circuits. For example, blockade of accumbal D2 dopamine receptors selectively inhibits lever-pressing for ethanol (motivational behavior) but has no effect on ethanol consumption if the lever-press response requirement is removed (Samson and Chappell, 2004). Therefore, shifting dopamine transmission into a phasic mode may primarily promote ethanol seeking behaviors without significantly altering the amount of ethanol consumed. Unfortunately, the intermittent drinking self-administration protocol employed in our present study does not provide a clear separation of seeking and consummatory drinking behaviors. Future experiments employing an operant drinking paradigm that procedurally separates these drinking behaviors will be needed to more rigorously evaluate the role of phasic dopamine release on motivational aspects of ethanol seeking.

### Conflict of interest statement

The authors declare that the research was conducted in the absence of any commercial or financial relationships that could be construed as a potential conflict of interest.
